# Boreal and Lusitanian species display trophic niche variation in temperate waters

**DOI:** 10.1002/ece3.10744

**Published:** 2023-11-20

**Authors:** Morgane Amelot, Marianne Robert, Maud Mouchet, Dorothée Kopp

**Affiliations:** ^1^ Centre d'Ecologie et des Sciences de la Conservation UMR 7204 MNHN‐CNRS‐ Sorbonne Université, Muséum national d'Histoire naturelle de Paris Paris France; ^2^ UMR DECOD (Ecosystem Dynamics and Sustainability) IFREMER, INRAE, Institut Agro Plouzane France

**Keywords:** Celtic Sea, climate change, competition, habitat suitability, isotopes, thermal preferenda, trophic structure

## Abstract

Climate change has non‐linear impacts on species distributions and abundance that have cascading effects on ecosystem structure and function. Among them are shifts in trophic interactions within communities. Sites found at the interface between two or more biogeographical regions, where species with diverse thermal preferenda are assembled, are areas of strong interest to study the impact of climate change on communities' interactions. This study examined variation in trophic structure in the Celtic Sea, a temperate environment that hosts a mixture of cold‐affiliated Boreal species and warm‐affiliated Lusitanian species. Using carbon and nitrogen stable isotope ratios, trophic niche area, width, and position were investigated for 10 abundant and commercially important demersal fish species across space and time. In general, the niches of Boreal species appear to be contracting while those of Lusitanian species expand, although there are some fluctuations among species. These results provide evidence that trophic niches can undergo rapid modifications over short time periods (study duration: 2014–2021) and that this process may be conditioned by species thermal preferenda. Boreal species displayed spatial variation in trophic niche width and seem to be facing increased competition with Lusitanian species for food resources. These findings underscore the need to utilize indicators related to species trophic ecology to track the ecosystem alterations induced by climate change. Such indicators could reveal that the vulnerability of temperate ecosystems is currently being underestimated.

## Graphical abstract

A contraction of the Boreal species niche and, reversely, an expansion of the Lusitanian species niche were observed in the Celtic Sea. These findings provided among the first evidence of fast trophic niche modifications conditioned by species thermal affinities and, as such, highlighted this ecosystem's potential vulnerability to climate change.

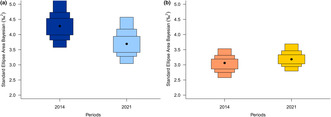

## 1 INTRODUCTION

Marine ecosystems worldwide are responding to climate change at various biological scales, from organisms to communities (Koenigstein et al., 2016; Scheffers et al., 2016). These impacts are not experienced equally by all marine species, resulting in the reorganization of species interactions and ecosystem structure. Indeed, the magnitude and speed of global warming's influence are locally dependent on marine environment spatial heterogeneity (e.g., currents and water stratification), which results in differential species exposure to climate change (García Molinos et al., 2017; Pinsky et al., 2013). Furthermore, species are reacting to climate change in a way that is shaped by their vulnerabilities, which are determined by their habitats spatial continuity and dispersal barriers (Keith et al., 2011; McHenry et al., 2019), as well as their life history traits such as their mobility and thermal preferenda (Sunday et al., 2015). Because species differ in their vulnerability (i.e., degree of exposure and sensitivity) to climate change, the result is non‐linear and hardly predictable changes in ecosystem structure and function (Johnson et al., 2011; Williams et al., 2008).

The world's biogeographical regions will experience global warming differently (e.g., Arctic Amplification, Previdi et al., 2021), which should lead to different responses in species diversity, abundance, and interactions. Polar regions are predicted to experience important changes in species composition, given that species will not be able to move to higher latitudes to remain in the same water temperature range. Tropical waters are expected to suffer from high levels of local extinction, given that many native species have a very narrow window of thermal tolerance whose upper limit is close to the habitat's maximum temperature (Cheung et al., 2009; Doney et al., 2012). Thus, temperate waters are often considered to be less vulnerable regions. However, areas found at the interface of two or more biogeographical regions are areas where species with a range of thermal preferenda are assembled. Some occur at their southern distribution limit, while others occur at their northern distribution limit. Species might be acclimated to broader temperature ranges (McKenzie et al., 2021; Sandersfeld et al., 2017), which means their biodiversity and abundance could be less impacted by global warming compared to those of species in regions where thermal conditions are more homogenous. As such, these areas present a unique opportunity for studying the impact of climate change on species and community interactions. Moreover, future temperature changes in these areas are likely to benefit at least one of the biogeographic communities present (Rijnsdorp et al., 2009), and the resulting increase in diversity or abundance could mask adverse effects on other biogeographical groups (Elahi et al., 2015; Hillebrand et al., 2018). Additionally, stability in species composition does not always translate into stability in species interactions, which could become reorganized as a consequence of slight changes in population sizes or species distributions (Harley et al., 2006). Structural changes are likely to be amplified in the near future as climate change has ever‐larger impacts on species diversity and abundance. Indeed, species are moving poleward at faster speeds in marine than terrestrial ecosystems (i.e., 72 ± 13.5 km dec^−1^ against 6.1 ± 2.4 km dec^−1^; Poloczanska et al., 2013, 2016). At a finer spatial scale, these areas located at the interface between two or more biogeographical regions, especially in temperate waters like the Celtic Sea, might crystallize impacts on species and interactions between biogeographical groups.

At higher biological scales, climate change will likely modify species distributions in such a way that both species and their trophic interactions will be affected. The study of trophic niche variations allows for the determination of changes in predator interactions with their prey as well as with their competitors (Fuhrmann et al., 2017; Gulka et al., 2017). Climate change has diverse impacts on trophic niches, from shifting their position (displacement) or their width (expansion/contraction) to altering their relationships with each other (segregation/overlap) (Kingsbury et al., 2020). For instance, Kingsbury et al. (2020) studied variability in the niches of tropical and temperate fish species along a latitudinal gradient in the Southern Hemisphere. It was found that in regions where species of both thermal affinities co‐occurred, tropical species exhibited their largest niche area at the lowest latitudes, where the temperate niche area was the smallest, and conversely, temperate species niche area was the largest at higher latitudes, where the tropical niche area was the smallest. Trophic niche variation arising from environmental changes (e.g., niche width contraction or expansion) might also be occurring in a given area over time (Bond & Lavers, 2014; Ogilvy et al., 2022). Such spatial and temporal shifts are expected to become more frequent in the coming years as the impacts of climate change increase in magnitude and influence prey and predator alike.

The Celtic Sea is a temperate sea that gathers species with different biogeographic affinities linked to their thermal preferenda. It hosts Boreal species, generally found in colder waters, and Lusitanian species, generally found in warmer waters (Engelhard et al., 2011). In this area, climate change influence on species abundance and composition has already been investigated, but the effects on ecosystem trophic structure have yet to be studied (Eme et al., 2022; Hernvann et al., 2020; Lauria et al., 2012; Mérillet et al., 2020; Ter Hofstede et al., 2010). Isotopic niches investigation through the analysis of δ^13^C and δ^15^N stable isotope signatures offers an integrative reflect of species dietary patterns over intermediate time periods that overcome daily‐based prey availability (Layman et al., 2007; Nielsen et al., 2018). In the temperate waters of the Northern Hemisphere, climate change is expected to cause trophic niche contractions for Boreal species and trophic niche expansions for Lusitanian species. Several factors may be behind niche contractions. First, warmer water temperatures may result in an increased abundance of Lusitanian species, augmenting the predation pressure exerted by these species and, consequently, decreasing the prey available to Boreal species through competition for food (Lancaster et al., 2017). Second, niche contraction could be caused by a reduction in suitable habitat surfaces (Kitchel et al., 2022). The density of Boreal predator species might decline at sites that are less thermally suitable than in the past, causing niche contraction at these locations (Riverón et al., 2021). Areas offering distinct feeding opportunities might also become less suitable for Boreal species, triggering the inaccessibility of particular prey species (Tunney et al., 2014). Finally, and more broadly, as for predators, the spatial distribution and abundance of prey could also be affected by climate change, which would restrict their accessibility to predators within a given area (Durant et al., 2019).

This study thus explored whether trophic niche width and position changed for fish species in an area gathering species with various biogeographic affinities over a relatively short time period (2014–2022), taking into account species thermal preferenda and depth.

## 2 MATERIALS AND METHODS

### 2.1 Data collection and processing

**FIGURE 1:**
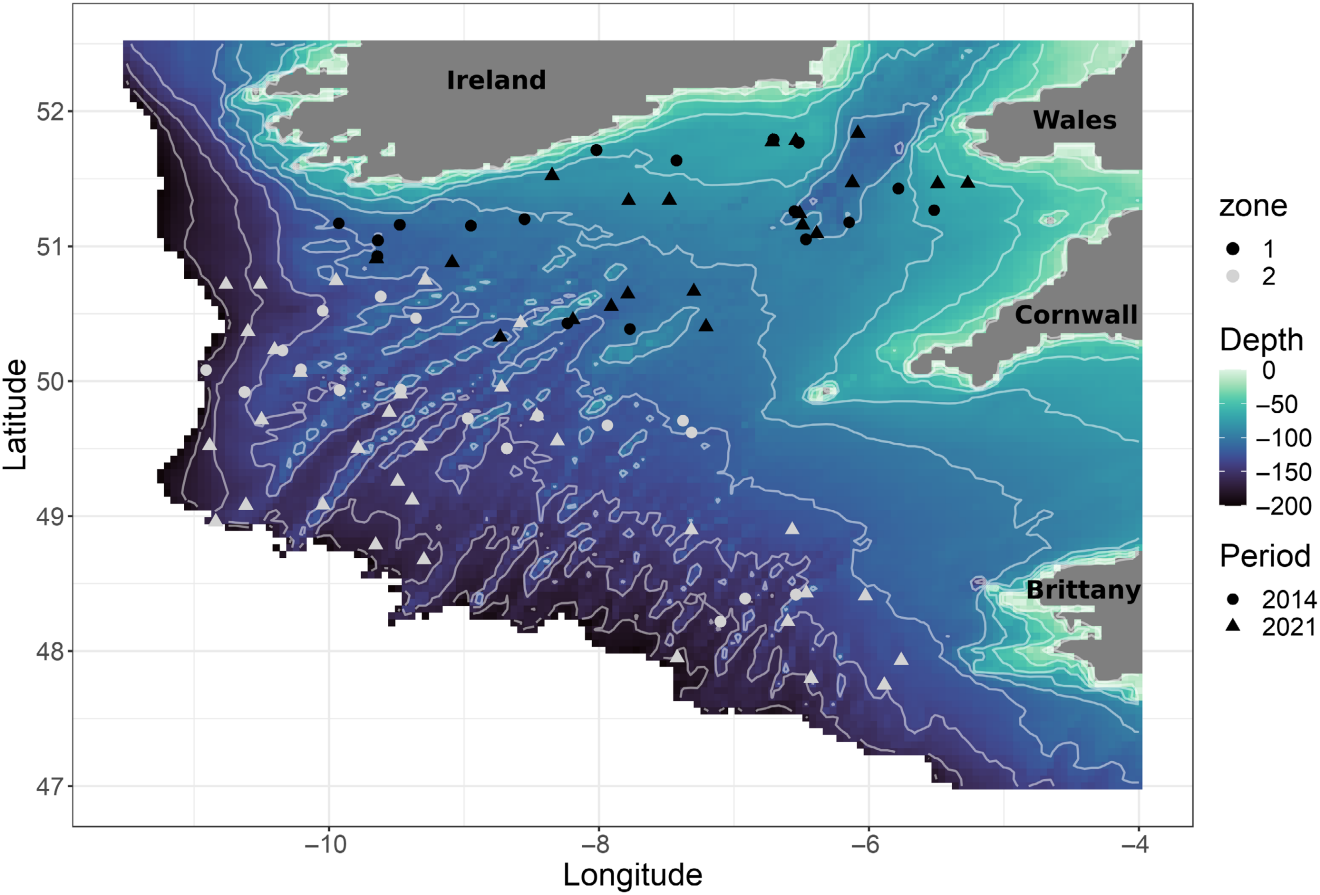
Sampling locations in 2014 (circles) and 2021 (triangles) for Zone 1 (in black) and Zone 2 (in gray). The white lines are the 20‐m bathymetric isoclines.

Four Boreal species (cod: *Gadus morhua*, haddock: *Melanogrammus aeglefinus*, blue whiting: *Micromesistius poutassou*, and plaice: *Pleuronectes platessa*) and six Lusitanian species (megrim: *Lepidorhombus whiffiagonis*, black bellied angler: *Lophius budegassa*, European angler: *Lophius piscatorius*, whiting: *Merlangius merlangus*, hake: *Merluccius merluccius*, and sole: *Solea solea*) were sampled in the Celtic Sea during the EVHOE campaign (*EValuation des ressources Halieutiques de l'Ouest de l'Europe*), which was part of the International Bottom Trawl Survey (Duhamel et al., 2014; Laffargue et al., 2021, 2022; Leaute et al., 2015, 2016). This campaign took place in November and December. Samples were collected with a GOV (Grande Ouverture Verticale) demersal trawl, which was towed for 30 min at a mean speed of 3.5 knots by R/V Thalassa. Sampling took place in two different zones (Day et al., 2019): Zone 1 was coastal and had a maximum depth of 127 m, and Zone 2 was farther out to sea and had a maximum depth of 177 m (Figure 1). The objective was to sample 10 individuals per species per zone; however, given the reality of species spatial distributions, achieving this target was not always possible (Appendix S1).

A total of 553 fish and 61 primary consumers (*Pecten maximus*) were collected over two time periods (Appendix S1): a 3‐year span from 2014 to 2016 (hereafter 2014, Robert et al., 2022) and a 2‐year span from 2021 to 2022 (hereafter 2021). The primary consumers were used to establish the isotopic baseline.

White dorsal muscle was collected from the fish, and abductor muscle was collected from the bivalve primary consumers. All samples were kept frozen until they could be processed in the laboratory. Then, they were oven dried (60°C for 48 h) and ground into a homogeneous powder using a mixer mill. Their stable isotope ratios of carbon (^13^C/^12^C, hereafter δ^13^C) and nitrogen (^15^N/^14^N, hereafter δ^15^N) were determined by the Stable Isotopes in Nature Laboratory (University of New Brunswick, Canada) using a Carlo Erba NC2500 Elemental Analyzer.

### 2.2 Data analysis

#### 2.2.1 Baseline correction

The δ^13^C and δ^15^N values were corrected using bivalves (i.e., the ecosystem's primary consumers) as the baseline (Day et al., 2019). A suspension‐feeding bivalve, the king scallop *Pecten maximus*, was chosen as the trophic baseline for this study (Barnes et al., 2009; Jennings & Warr, 2003). Using a primary consumer as a baseline offers the advantage over primary producers, such as phytoplankton, of buffering short‐term variations in isotopic value (e.g., seasonality in environmental factors). Isotopic values of the trophic baseline, and thus of species at higher trophic levels, may vary spatially due to environmental gradients, such as depth. It results in the observation that the isotopic values of higher‐trophic level species must be corrected for spatial variation in baseline values. The following equation, which incorporated information about primary consumer sample depth, was employed (Day et al., 2019):

(1) $$\delta Xi_{\text{corrected}}\;=\delta Xi_{\text{uncorrected}}\;–\left(\alpha^{*}\;{\text{depth}}_{\delta \mathrm{Xi}}+\beta \right)+\text{mean}\;\left(\delta X_{P.\text{maximus}\;\text{in Celtic}\;\mathrm{Sea}}\right)$$

where δ*X* is the *X* isotope ratio for a given individual *i*; *α* and *β* are the coefficients estimated from the fitted linear regression model; depth_δ*Xi*_ is the depth at which individual *i* was collected; and mean (δ*X*_*P.maximus* in Celtic Sea_) is the mean *X* isotope ratio of *Pecten maximus* across the Celtic Sea. The linear correlation between δ^15^N and δ^13^C ratio and depth was estimated based on the Pearson correlation coefficient, respectively −0.925 (*p* < .001) for the δ^15^N ratio and −0.849 (*p* < .001) for the δ^13^C ratio. Assumptions related to the linear regression, normality (Shapiro test, δ^15^N *p* = .560, δ^13^C *p* = .880), and homoscedasticity (Breusch‐Pagan test, δ^15^N *p* = .551, δ^13^C *p* = .051) were verified.

#### 2.2.2 Trophic niche width and overlap

Trophic niche width and overlap were analyzed at the level of (1) the thermal preferenda group (Boreal versus Lusitanian) and (2) species. Trophic niche width was compared across time periods for both groups and species by zone, except in the case of *Solea solea* in Zone 2 because the sample size was too small. Trophic niche overlap was compared across time periods exclusively between thermal preferenda groups.

Here, the standard ellipse area (SEA) encompassed a defined percentage of a species or group displaying a given isotopic signature; 95% SEA for the δ^15^N and δ^13^C signatures were used. SEA simultaneously represents the width and position of a species' or group's isotopic niche in two‐dimensional space (i.e., a surface area expressed in ‰^2^). If SEA position changes in δ^13^C, it suggests that basal prey species or groups varied, as δ^13^C typically increased by ~1‰ between predator and prey (Grippo et al., 2011; Post, 2002). If SEA position changes in δ^15^N, it suggests that there has likely been a change in prey trophic level (Layman et al., 2007). More specifically, δ^15^N increases by ~3.4‰ on average between predator and prey (Newsome et al., 2007; Post, 2002). A decreased SEA width indicates that the size of a species' or group's isotopic niche has contracted, whereas an increase in SEA width indicates that the size of a species' or group's isotopic niche has expanded.

Bayesian Standard Ellipse Area (hereafter SEA.B) corresponds to a particular calculation method for SEA that allows for robust statistical comparisons among data sets with different sample sizes (Jackson et al., 2011). SEA.B values were calculated using the *siberEllipses()* function by thermal preferenda group and species for each zone and time period (2014, 2021), and the convergence of the model's posterior distributions was checked using Gelman's test. The probability that SEA.B width varied over time by group and species were calculated and compared.

Finally, the degree of resource competition between the Lusitanian and Boreal groups was estimated by examining niche overlap. SEA.B overlap was simulated for the two time periods (*n* = 1000 samples, *maxLikOverlap()* function, with Boreal and Lusitanian groups as community variables and species as the group variables) and statistically compared using a Student's *t*‐test.

To better visualize the variation in Bayesian overlap variations, the values of the latter were displayed employing 95% ellipses. All analyses were conducted using R (v. 4.2.2; R Core Team, 2016) and the SIBER package (Jackson et al., 2011).

## 3 RESULTS

### 3.1 Niche contraction and expansion

**FIGURE 2:**
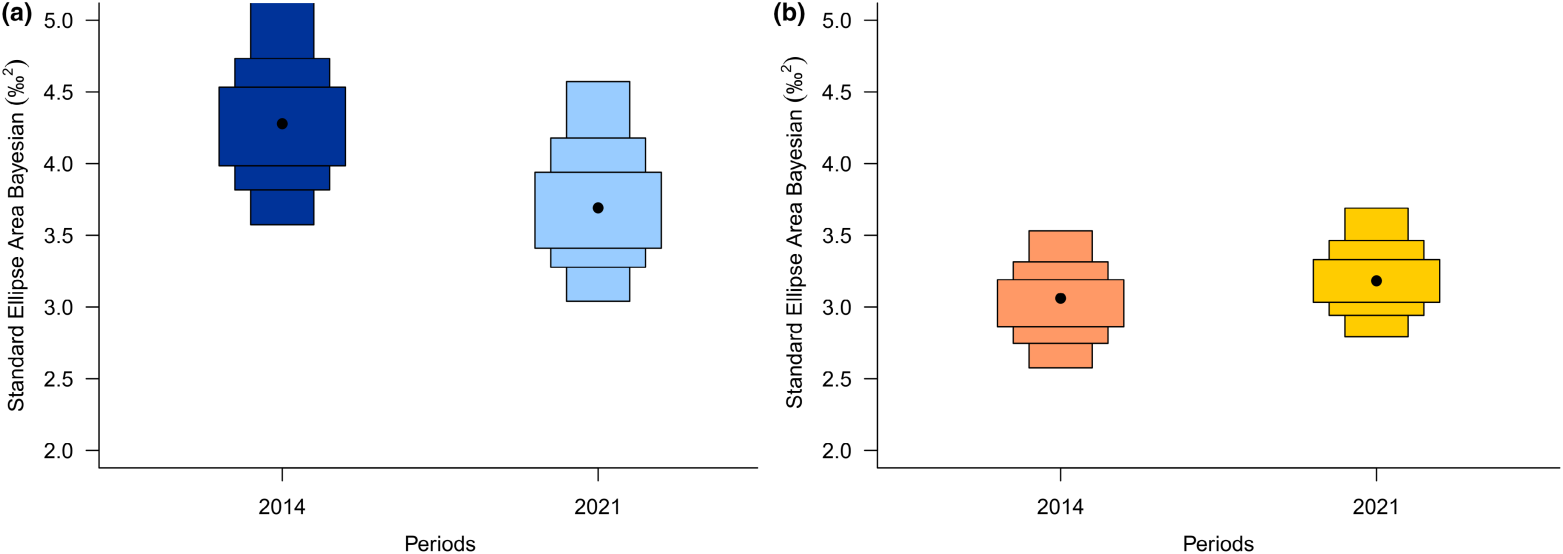
Bayesian Standard Ellipse Area values (in ‰^2^) for the (a) Boreal and (b) Lusitanian thermal preferenda groups in 2014 and 2021.

Between the two time periods, the SEA.B of the Boreal group decreased (mean: 4.34‰^2^ in 2014 vs. 3.77‰^2^ in 2021; probability of decrease: 84.2%), whereas the SEA.B of the Lusitanian group increased (mean: 3.05 ‰^2^ in 2014 vs. 3.22 ‰^2^ in 2021; probability of increase: 69.93%) (Table 1, Figure 2). The δ^15^N mean range of the SEA.B increased for the Boreal group (mean range: 2.86 in 2014 vs. 3.63 in 2021), but decreased for the Lusitanian group (mean range: 1.56 in 2014 vs. 1.21 in 2021). In contrast, the mean δ^13^C range from SEA.B decreased for the Boreal group (1.93 in 2014 vs. 1.06 in 2021) and increased for the Lusitanian group (1.27 in 2014 vs. 1.90 in 2021).

**TABLE 1:** Bayesian Standard Ellipse Area (SEA.B) values (in ‰^2^) for each thermal preferenda group and species in the time periods 2014 and 2021.

Thermal preferenda group/species	Whole Celtic Sea			Zone 1			Zone 2		
	SEA.B 2014	SEA.B 2021	*p* (%)	SEA.B 2014	SEA.B 2021	*p* (%)	SEA.B 2014	SEA.B 2021	*p* (%)
**Boreal**	4.32	3.77	84.2	3.37	3.46	43.1	5.03	4.54	67.8
*G. morhua*	1.14	1.62	11.9	0.91	1.51	9.5	0.65	1.26	7.7
*M. aeglefinus*	2.96	2.14	90.8	1.41	2.10	8.6	2.48	1.15	96.1
*M. poutassou*	2.12	2.29	42.1	1.76	2.73	15.4	0.53	3.32	<1
*P. platessa*	1.77	0.82	>99	2.25	0.80	>99	0.24	0.77	4.8
**Lusitanian**	3.05	3.22	30.1	2.47	3.10	5.8	3.31	3.05	67.8
*L. whiffiagonis*	0.93	1.86	<1	0.73	1.15	11.3	1.21	1.32	39.4
*L. budegassa*	1.39	2.16	5.4	0.54	3.15	<1	2.32	1.09	97.6
*L. piscatorius*	4.44	5.48	21.7	2.96	3.89	23.5	5.46	4.56	69.1
*M. merlangus*	1.43	1.50	42.9	1.19	1.02	68.7	0.48	0.98	5.4
*M. merluccius*	1.76	1.63	62.8	1.11	1.02	62.7	1.62	0.73	98.7
*S. solea*	1.17	1.93	10.8	1.01	1.10	40.0	‐	‐	‐

*Note*: Also included are the probabilities that SEA.B decreased between time periods, both for the entire Celtic Sea and for each zone. Probabilities corresponded to the probability of decreasing the SEA.B along time. Consequently, probabilities have the following meaning: values above 50% indicate that SEA.B likely decreased between time periods (light gray), while values below 50% indicate that SEA.B likely increased between time periods (dark gray).

**FIGURE 3:**
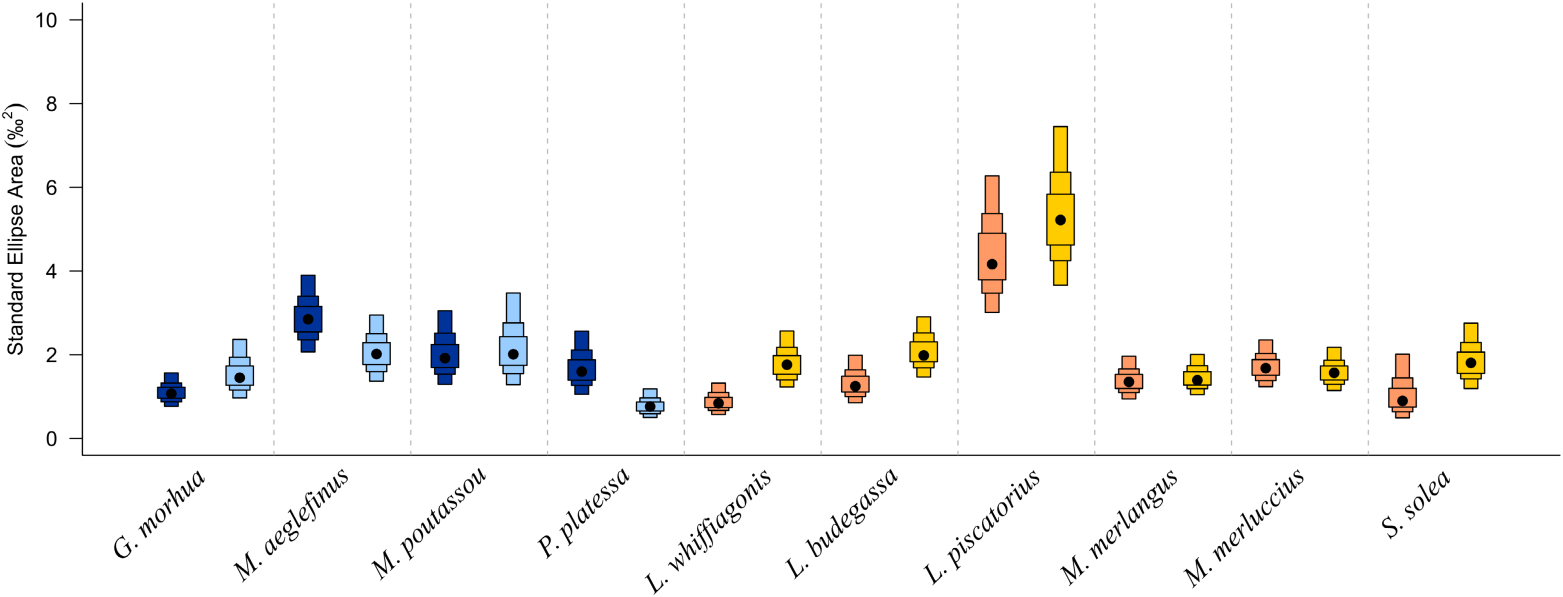
Bayesian Standard Ellipse Area values (in ‰^2^) for the Boreal species in 2014 (dark blue) and 2021 (light blue) and for the Lusitanian species in 2014 (orange) and 2021 (yellow).

At the species level, there were pronounced drops in the SEA.B values of two Boreal species, *Pleuronectes platessa* (1.77‰^2^ in 2014 vs. 0.82‰^2^ in 2021; probability >99%) and *Melanogrammus aeglefinus* (2.96‰^2^ in 2014 vs. 2.14‰^2^ in 2021; probability of 90.8%), rise in the SEA.B values of all the Lusitanian species, with the exception of *Merluccius merluccius*. The largest increases were seen for *Lepidorhombus whiffiagonis* (0.93‰^2^ in 2014 vs. 1.86‰^2^ in 2021; probability >99%) and *Lophius budegassa* (1.39‰^2^ in 2014 vs. 2.16‰^2^ in 2021; probability of 94.6%) (Table 1, Figure 3).

### 3.2 Niche overlap

**FIGURE 4:**
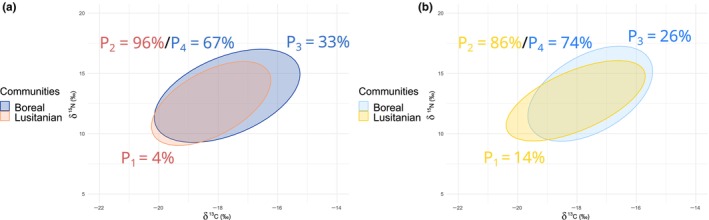
95% Bayesian Standard Ellipse Area (SEA.B) values for both the Boreal and Lusitanian thermal preferenda groups in (a) 2014 and (b) 2021. P_1_, percentage of Lusitanian SEA.B that does not overlap with Boreal SEA.B; P_2,_ percentage of Lusitanian SEA.B that does overlap with Boreal SEA.B; P_3_, percentage of Boreal SEA.B that does not overlap with Lusitanian SEA.B; P_4_, percentage of Boreal SEA.B that does overlap with Lusitanian SEA.B.

Between 2014 and 2021, the overlap between the Boreal and Lusitanian isotopic niches increased. The proportion of overlap over the total SEA.B of the two groups (*n* = 1000) increased by 1% on average, from 64% in 2014 to 65% in 2021 (*p* < .001). The proportion of the Boreal trophic niche overlapped by the Lusitanian trophic niche increased through time, from 67% in 2014 to 74% in 2021. Contrarily, the proportion of the Lusitanian trophic niche overlapped by the Boreal trophic niche decreased over time, from 96% in 2014 to 86% in 2021 (Figure 4).

### 3.3 Spatial patterns

Between the two time periods, the isotopic niche of the Lusitanian group grew in Zone 1 (2.47‰^2^ in 2014 vs. 3.10‰^2^ in 2021; probability of increase: 94.2%). However, few changes were observed in either zone for the Boreal group (Table 1).

At the species level, changes occurred in both zones. In the Boreal group, a decrease was seen in SEA.B for *Pleuronectes platessa* in Zone 1. In the Lusitanian group, an increase in SEA.B was observed for *Lophius budegassa* SEA.B in Zone 1. Various shifts occurred in Zone 2. In the Boreal group, SEA.B increased for *Micromesistius poutassou* and *P. platessa*, while it decreased for *Melanogrammus aeglefinus*. For Lusitanian species, SEA.B decreased for *L. budegassa* and *Merluccius merluccius* (Table 1).

## 4 DISCUSSION

Despite the short time period covered by the data used in this analysis (<10 years) compared to similar studies on isotopic niche variations along time (Bond & Lavers, 2014; Ogilvy et al., 2022; Ólafsdóttir et al., 2021), changes were seen in trophic niche width and position for both Boreal and Lusitanian fishes. These results suggest that certain areas, such as the temperate zone at the boundary between Lusitanian and Boreal biogeographical areas, may be particularly sensitive to global changes. These changes in 10 predator species trophic niches are likely to result in, or to be the result of, deep changes in Celtic Sea ecosystem structure and functioning through cascading effects on lower trophic levels.

Previous research has documented the contraction and expansion of species trophic niche widths in relation to environmental conditions (e.g., temperature range, levels of primary productivity, habitat degradation) across latitudinal and temporal gradients (Bond & Lavers, 2014; Kingsbury et al., 2020). This study's findings suggest that the thermal preferenda of fish may condition trophic niche variation, even if there is some interspecific fluctuation. The trophic niche of the Boreal group contracted between time periods, while that of the Lusitanian group expanded.

Three complementary, non‐mutually exclusive hypotheses could explain the observed spatiotemporal changes in isotopic niches: (i) an increase in food competition between the biogeographical groups that favored the Lusitanian species; (ii) a shift in habitat suitability that could have had two main consequences. On one side, a shift in habitat suitability could have caused modifications in predator densities. A change in predator density could have led to more diverse predator diets in areas with higher predator densities and less diverse predator diets in areas with lower predator densities. On the other hand, shifts in habitat suitability could have impacted the diversity of prey available to predators, resulting in different diets when predators shifted to new habitats; (iii) a modification in the distribution and abundance of prey communities.

Boreal and Lusitanian species displayed trophic niche overlap, indicating that competition for food resources exists between the two biogeographical groups (Gaspar et al., 2022; Pankow et al., 2021; Pelage et al., 2022). The surface area of the Boreal group trophic niche overlapped by the Lusitanian group increased, while the surface area of the Lusitanian group trophic niche overlapped by the Boreal group decreased. One interpretation of these results is that the Lusitanian species were exerting greater competitive pressure on the Boreal species, while the Boreal species were exerting less competitive pressure on the Lusitanian species. This interpretation is consistent with the observed decrease and increase in the δ^13^C range for the Boreal and Lusitanian groups, respectively. δ^13^C is usually an indicator of the food resources exploited by a given consumer (France, 1995). The co‐variation of the Boreal and Lusitanian groups δ^13^C might indicate that competition was restricting the access of Boreal species to specific resources. Potential competitive exclusion was also observed at the species level in the shifted trophic niches of flatfish (*Pleuronectes platessa* and *Lepidorhombus whiffiagonis*), with *P. platessa* displaying a decrease in trophic niche width and *L. whiffiagonis* displaying an expansion. These species share certain prey, mainly crustaceans (e.g., Caridea, Mysida, Brachyura, Eucarida), which lends support to the idea that competition for certain prey might have increased (Rault et al., 2017).

The Boreal group's niche contraction supports the hypothesis that current temperatures in the Celtic Sea are no longer as suitable for these fish as they were in the past. Niche contraction is seemingly a signal of the biogeographical group's response to climate change. In the Arctic Ocean, changes in environmental conditions have led to distribution shifts of Arctic and Boreal fish communities and to trophic structure reorganization that favored Boreal over Artic fish communities (Fossheim et al., 2015; Kortsch et al., 2015). Moreover, the observed changes in trophic niche width had a spatial component. In the deeper Zone 2, increases were seen in the trophic niches of all the Boreal species, apart from *Melanogrammus aeglefinus*. Marine species are not only engaging in poleward movement. They are also heading to greater depths, which could act as thermal refuges for cold‐affiliated species (Dulvy et al., 2008; Perry et al., 2005; Pinsky et al., 2013). The fact that Boreal species expanded their trophic niches in Zone 2 could indicate that deeper waters now provide more suitable habitat. In coastal Zone 1, the greatest trophic shift was observed for *Pleuronectes platessa*, whose niche substantially contracted in width. This species is more coastal than the other Boreal species considered in this study, and coastal waters are more likely to experience faster and higher temperature variations than deeper waters (Harley et al., 2006). Indeed, a large proportion of coastal zones might no longer fit with the temperature preferenda of *P. platessa*. Such changes in habitat and zone suitability might be responsible for the Boreal species' observed niche expansion into deeper waters and *P. platessa*'s niche contraction in the coastal zone. It might be that higher densities of a particular predator species could result in higher levels of individual dietary specialization, which could lead to, in turn, a more diverse range of individual diet specialization within the population, prompting broader niche sizes at the predator species level. On the flip side, a decrease in the density of a predator species could result in less diverse intraspecific predator diets and, as a consequence, trophic niche contraction (Riverón et al., 2021). This hypothesis should be further explored by comparing the densities and trophic niche widths of fish species over time and space. The loss of suitable habitats could also have resulted in *P. platessa* losing access to certain prey types in coastal zones. At the same time, by gaining access to suitable habitats in the depths, Boreal species may have also gained access to new prey (Davis et al., 2022; Selden et al., 2018; Tunney et al., 2014).

Climate change may also be directly impacting prey distributions, diversity, and abundance. Past research has observed that benthic invertebrates in the North Sea are moving both poleward and downward but at a speed that is not keeping pace with climate change; as a result, the abundance and diversity of prey species could be declining (Hiddink et al., 2015). Comparable prey distribution shifts could be occurring in the Celtic Sea and might have played a role in the changes in predator niche width seen in this study. Finally, prey diversity and abundance could be impacted directly or indirectly by increases in prey mortality as a result of dedicated fisheries, habitat disruptions (e.g., wind farms, aggregate dredging), or pollution (e.g., microplastics, heavy metals) (Collie et al., 2017; Halpern et al., 2008, 2015; Hiddink et al., 2016).

Climate change is expected to give rise to tropicalization, defined as an increase in warm‐affiliated species, and to deborealization, defined as a decrease in cold‐affiliated species. Deborealization results in a larger loss of abundance and biodiversity and has been shown to primarily affect areas with high levels of thermal diversity (McLean et al., 2021). Several authors have advocated that shifts in species distributions will lead to the reorganization of ecosystem trophic structure (Durant et al., 2019; Pinsky et al., 2020). This study supports the above hypothesis by providing some of the first evidence that trophic structures may reorganize themselves rapidly in response to environmental changes, which could be seen in the negative effects on the Boreal group. The association between variation in trophic niche size and species thermal preferenda strongly supports the idea that climate shifts will influence ecosystem structure. As a result, it is of primary interest to monitor species trophic ecology to pick up on any alterations in food web structure that might be amplified by climate change in the near future.

## AUTHOR CONTRIBUTIONS

**Morgane Amelot:** Conceptualization (equal); formal analysis (equal); writing – original draft (equal); writing – review and editing (equal). **Marianne Robert:** Conceptualization (equal); funding acquisition (equal); supervision (equal); writing – original draft (equal); writing – review and editing (equal). **Maud Mouchet:** Conceptualization (equal); funding acquisition (equal); supervision (equal); writing – original draft (equal); writing – review and editing (equal). **Dorothée Kopp:** Conceptualization (equal); funding acquisition (equal); supervision (equal); writing – original draft (equal); writing – review and editing (equal).

## Supporting information


Appendix S1

## Data Availability

The data that support the findings of this study are derived from the following resources available in the public domain (Robert et al., 2022).
