# Supplementary material for: Boreal and Lusitanian species display trophic niche variation in temperate waters
Source: Ecol Evol. 2023 Nov 20;13(11):e10744. doi: 10.1002/ece3.10744 (PMC10659821; doi:10.1002/ece3.10744)
Supplement: Supplementary file 1 [file ECE3-13-e10744-s001.odt]

Supplementary Material and methods 1 : Isotopes data collect and analysis

Table 1 Number of isotopic samples by species in each zone for each period (COD : Gadus morhua, HAD : Melanogrammus aeglefinus, WHB : Micromesistius poutassou, PLE : Pleuronectes platessa, MEG : Lepidorhombus whiffiagonis, ANK : Lophius budegassa, MON : Lophius piscatorius, WHG : Merlangius merlangus, HKE : Merluccius merluccius, SOL : Solea solea

|  | Species |
| --- | --- |
| COD | HAD | WHB | PLE | MEG | ANK | MON | WHG | HKE | SOL |
| Thermal preferendum | Boreal | Lusitanian |
| Period | Zone |  |  |  |  |  |  |  |  |  |  |
| 2014 | Zone 1 | 18 | 25 | 12 | 14 | 13 | 12 | 16 | 22 | 23 | 7 |
| Zone 2 | 14 | 14 | 10 | 7 | 10 | 11 | 13 | 8 | 16 | 2 |
| Whole Celtic Sea | 32 | 39 | 22 | 21 | 23 | 23 | 29 | 30 | 39 | 9 |
| 2021 | Zone 1 | 13 | 20 | 10 | 19 | 15 | 16 | 19 | 24 | 22 | 15 |
| Zone 2 | 8 | 8 | 7 | 4 | 15 | 19 | 15 | 13 | 17 | 7 |
| Whole Celtic Sea | 21 | 28 | 17 | 23 | 30 | 35 | 34 | 37 | 39 | 22 |
